# Design and Mechanical Characterization of Voronoi Structures Manufactured by Indirect Additive Manufacturing

**DOI:** 10.3390/ma13051085

**Published:** 2020-02-29

**Authors:** Daniele Almonti, Gabriele Baiocco, Vincenzo Tagliaferri, Nadia Ucciardello

**Affiliations:** 1 Dipartimento di Ingegneria dell’Impresa “Mario Lucertini”, Università di Roma “Tor Vergata”, Via del Politecnico 1, 00133 Rome, Italy; daniele.almonti@uniroma2.it (D.A.); tagliafe@uniroma2.it (V.T.); 2 Dipartimento di Ingegneria, Università degli Studi Roma Tre, Via Vito Volterra 62, 00146 Rome, Italy; gabriele.baiocco@uniroma2.it

**Keywords:** metal foam, additive manufacturing, open cell, compression test, random structure

## Abstract

Additive manufacturing (AM) is a production process for the fabrication of three-dimensional items characterized by complex geometries. Several technologies employ a localized melting of metal dust through the application of focused energy sources, such as lasers or electron beams, on a powder bed. Despite the high potential of AM, numerous burdens afflict this production technology; for example, the few materials available, thermal stress due to the focused thermal source, low surface finishing, anisotropic properties, and the high cost of raw materials and the manufacturing process. In this paper, the combination by AM of meltable resins with metal casting for an indirect additive manufacturing (I-AM) is proposed. The process is applied to the production of open cells metal foams, similar in shape to the products available in commerce. However, their cellular structure features were designed and optimized by graphical editor Grasshopper®. The metal foams produced by AM were cast with a lost wax process and compared with commercial metal foams by means of compression tests.

## 1. Introduction

Additive manufacturing (AM) processes allow for the fast and accurate design of three-dimensional components. Complex geometries can be reproduced by overlayering several micro-metric tiers of metallic or polymeric materials. The dimension of the items in production can be directly obtained from a Computer-Aided Design (CAD) file [1,2]. Several technologies are now available for AM production. Mainly, they differ in layering process, operating principle, and suitable materials. In the Fused Head Modeling (FDM), a movable head extrudes a thin wire of thermoplastic polymer. Controlling the temperature after extrusion, the wire solidifies, creating a layer welded to the former. A wide range of cheap and nontoxic polymers can be extruded in an Fused Deposition Modeling (FDM) process, such as Acrylonitrile Butadiene Styrene (ABS), medical ABS, polyclactic acid (PLA), casting wax, and elastomers [3,4]. Despite its simplicity, inexpensiveness, and the raw materials rendered, the surface quality and accuracy are rather low compared to other technologies. However, FDM is still used for the production of low-cost and low added-value components [5]. The stereolithography (SLA) is an AM technology where a photosensitive resin after ultraviolet (UV) exposition hardens with a process called photopolymerization. In SLA process, a substrate immersed in a liquid resin is the base for the printing process. A low-power UV laser triggers the polymerization process, creating a solid layer on the substrate surface [6]. An SLA device requires adequate hardware and software control systems to achieve valuable results in terms of resolution and accuracy.

AM processes for metallic materials represent an interesting technology in manufacturing applications. The most applied AM processes for metals include laser beam melting (LBM), electron beam melting (EBM), and laser metal deposition (LMD). Metallic parts produced by AM are more suitable for industrial applications compared to polymeric parts. However, expensive machineries, as well as low surface finishing and demanding process settings, limit the application of these methodologies in industrial environments [7]. Metal foams are composed of biphasic and cellular materials, which combine good mechanical resistance with excellent thermal and acoustic properties [8]. In particular, high specific strength [9,10,11,12,13] and strain [14,15,16], excellent energy absorption [17,18], acoustic insulation [19], and heat dissipation media [20,21,22,23,24] make this class of materials increasingly useful for several multifunctional applications. The main problem afflicting metal foams regards the manufacturing process, and specifically the porosity distribution [25,26], as well as the connection with other components. The latter is a critical factor in structural and heat-exchange devices, because welding and brazing processes are time-consuming, costly, and not suitable for the most common materials exploited in metal foams production. This burdens their feasibility in industrial applications [27,28]. The cellular configuration design is fundamental, as its purpose is the definition of a commercial foam-like structure that matches specific application requirements. As a consequence of their random structure, the foams offer very valuable materials for filters [29,30,31] or heat exchange processes [32,33,34,35].

## 2. Materials and Methods

**Figure 1:**
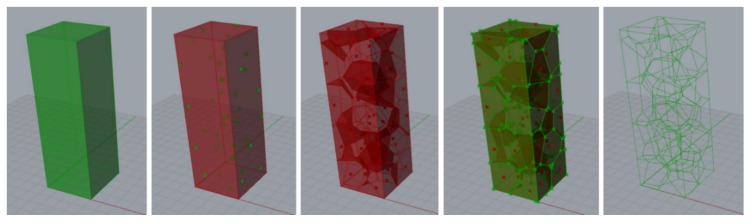
Process to achieve random structure in a space region by Voronoi tessellation [23].

The foam structure was realized using a method developed in a previous work [23]. The value that defines the distribution of porosities within a metal matrix is defined as pores per inch (PPI). It defines a linear distribution of pores; consequently, a specific volume distribution was defined as the cube of PPI value. A random distribution of points, corresponding to cell cores, was used to populate a reference volume. Consequently, the calculation of the points number was based on the designed metal foam value of PPI. A tessellation of the domain of interest was performed with a Voronoi division of space, *D* ∈${\mathbb{R}}^{3}$. Defined a number of points in *D*, $\left\{S_{i}\left(x_{i}\right)\right\}$ for i={1,…,N}, every point is associated a Voronoi cell, $C_{i}$, as follows:

(1) $$C_{i}\;=\;\left\{P\left(x\right)\in D|d\left(P,S_{i}\right)\leq d\left(P,S_{j}\right)\;\forall j\neq i\right\}$$

where d(⋆,⋆) is the Euclidean distance. The points corresponding to cores were identified randomly in a homogeneous distribution among the points generated. The cell individuated represents the space control of a point, specifically the part of domain adjacent to the nearest point. Various algorithms were proposed for realizing a Voronoi tessellation. In this paper, a cell-by-cell development procedure is presented. A cell ($C_{i}$ of point $S_{i}$) with the same dimensions of the whole region was defined. Subsequently, $C_{i}$ was modified by a recurring procedure, where other points ($S_{j}$) were examined by improving gap from $S_{i}$. After, each repetition (point $S_{j}$), $C_{i}$ was decreased to the convergence of the previously estimated pore and the portion of space closer to $S_{i}$ than $S_{j}$. This recursive procedure was stopped when the distance between point $S_{i}$ and point $S_{j}$ became adequate for the half-space closer to $S_{i}$ than $S_{j}$ to inevitably incorporate the entire cell. Equation (2) shows the isotropic criterion:

(2) $${\{\begin{array}{c} d\left(S_{j},S_{i}\right)>2d_{max}\\ d_{max}=max_{P\in C_{i}}d\left(P,S_{i}\right)=max_{P\in \left\{V_{i}\right\}}d\left(P,S_{i}\right) \end{array}}_{i}$$

where {*V_i_*} is the set of vertices of cell *C_i_*. The cells found in this process identified the spatial pattern. Convex polyhedral intersecting along flat faces was achieved. The process to obtain Voronoi structure was implemented as visual programming code inside Grasshopper® environment. The process applied to achieve the foam structure is reported in Figure 1. The volume of the foam sample was determined. Subsequently, tessellation process was performed; the pores were generated, creating a random cloud of points within the volume. These points determined the Voronoi cells; afterwards, the morphology exploited to produce the CAD model of the foam was obtained.

In the industrial applications, 5 and 10 PPI represent the common value of pores densities. In addition to these two densities, an intermediate value of 7 PPI was achieved. The number of pores used to obtain specific morphology was explained in Table 1.

**Figure 2:**
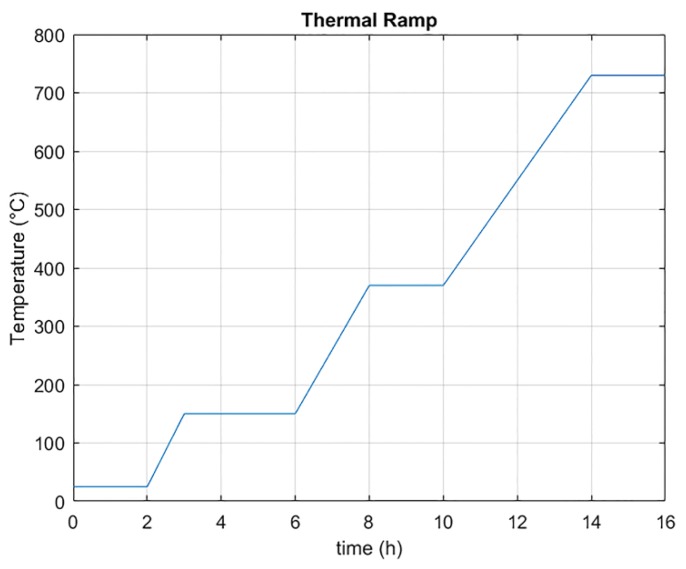
Thermal cycle exploited for shape preparation and model dewaxing.

Voronoi Tessellation enables individuation for each point of the set a cell. The individuated cell represents a portion of space corresponding to the influence region of a specific pore. The cell determines the region of space where all points of the space are closer to the core point than to any other. For each defined core, the convex cell was determined by axial planes of the segments that assemble it to the adjacent cores. Segments obtained from the reticular structure were thickened to 0.4 mm to achieve ligaments corresponding to commercial foams characteristics. Open surfaces and repeated entities were removed to achieve the printable model. Connecting rays were added in the junction between segments to achieve a structure comparable to commercial foams. The sacrificial pattern was obtained by a stereolithography 3D printer XFAB 2000 DWS (DWS, Thiene, Italy). The working area of the 3D printer is equal to a cylinder with a diameter of 180 mm. The machine utilizes a Solid State BluEdge® BE-1300X proprietary laser (DWS, Thiene, Italy), which permits achieving slicing in a range of 10–100 µm. The material exploited was FUSIA 444 (DWS, Thiene, Italy), which is a photosensitive resin for DWS 3D printers (DWS, Thiene, Italy), suitable for casting processes of elaborated models and thin details. The reticular structure of the CAD model helps avoid adding supports for the correct realization of the sacrificial pattern. The models obtained by additive procedure were situated in a UV oven for 1 h to complete the resin polymerization. Afterwards, the printed component was integrated in mold plaster (Ultra-Vest® MAXX, Ransom & Randolph, Maumee, OH, USA). The plaster powder was diluted in water at 25 °C, and the solution soaked and stirred at 316 rpm for 1 and 4 min, respectively. The mix was then poured into a foundry cylinder. With the aim of dissolving potential air inclusions, a vacuum machine and a vibrating platform were exploited in the plaster finishing. The plaster mold was dried at room temperature for 2 h, then the dewaxing process was exploited. Next, it was thermally treated. The thermal cycle, reported in Figure 2, provides for three heating ramps followed by a plateau. The thermal program is critical in order to remove material of sacrificial pattern from the mold and reach the casting temperature.

In parallel, the aluminum alloy EN43500 was heated to a temperature of 730 °C and mixed by low-frequency pulses before the casting. To realize the casting process, a molding device working in a vacuum condition was used. A casting temperature of 1,450 °C was reached with an accuracy of 4 °C. Following the casting process, the cylinder was cooled for six hours; subsequently, the plaster was opened. The casting achieved was cleaned from the pouring channels and the sprues. Then, the raw was machined to obtain a sample with dimensions of 20 × 20 × 40 mm, and specimens of comparable dimensions were produced from two typologies of commercial metal foam (m.pore® by MAYSER, (Mayser GmbH & Co KG, Lindenberg, Germany) and duocel® by ERG, (ERG Aerospace Corporation, Oakland, CA, USA). The commercial metal foams used in this work were both 10 PPI. Furthermore, both the commercial samples are produced with the same alloy exploited for the casting of the designed foams. Duocel® and m.pore® metal foams are realized by investment casting of polyurethane foam. In the former, the section of metal foam ligaments is variable in function of its relative density and process parameters. The duocel® metal foam ligaments section, achieved from the samples exploited in the present work, is triangular with the side pair of 0.4 mm, and the ligaments section of m.pore® metal foam is triangular with the side pair of 0.7 mm. To define the mechanical properties and compare the commercial models and designed foams, compression tests were realized. A standard mechanical tests device, “MTS 50 Insight” (MTS Systems Corporation, Eden Prairie, MN, USA, and a 50 kN load cell (MTS Systems Corporation, Eden Prairie, MN, USA) were used. The compression tests were realized under a constant deformation rate of 1 mm/min (quasi-static condition). As an end-test, condition was imposed: the achievement of 80% of deformation from the initial dimension. For each type, three specimens were prepared to verify the repeatability of the tests. From the stress-strain diagrams, it was possible to extrapolate the energy absorbed by the specific structures. The collapse stress “σ” was evaluated as a ratio between force F (N) and an apparent cross-sectional area A (mm^2^) of the sample

(3) $$\sigma =\frac{F}{A}\;\left(MPa\right)$$

Strain ε (%) was calculated from the deflection L (mm) and initial height h (mm) of the specimen

(4) $$\varepsilon =\frac{L}{h}\cdot 100\;\left(\%\right)$$

Deformation energy absorbed W (J) needed for deformation up to L

(5) $$W=\int_{0}^{L}F\left(L\right)dL$$

## 3. Results and Discussion

By exploiting the algorithm that had been developed, reticular random structures were realized. The nature of the cellular structure made the foam self-supporting. Therefore, the application supporting elements during the foam model manufacturing was redundant and unnecessary.

In contrast to the commercial foams, the morphology achieved shows the number of cells required in order to be classified with the correct PPIs number. The cell number of the designed foam is required to allow the classification in the theoretical PPI number; in this way, CAD models are completely controllable as parameters. During the casting, while the structure thickened and the PPI increased, no damage in the gypsum shape was produced, confirming the goodness of the thermal cycle chosen.

**Figure 3:**
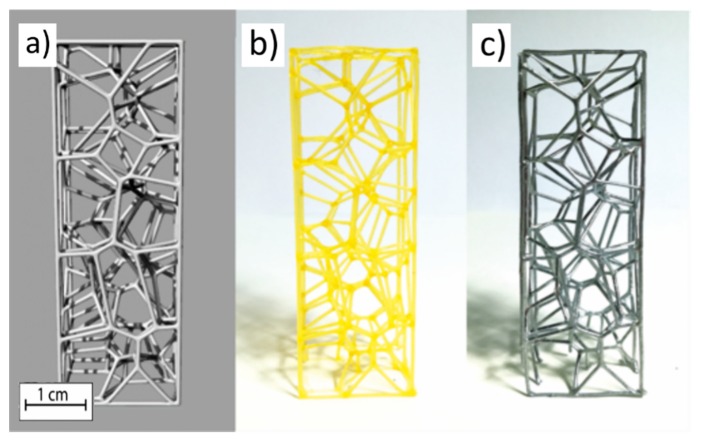
Comparison between (**a**) CAD model, (**b**) printed model, and (**c**) specimen obtained from foundry [23].

Figure 3 highlights the similarity between the CAD model, AM model, and cast specimen. It shows the compliance of structure carried out in the various sub-processes. Therefore, there is full control of structural parameters, such as the number of cells and ligaments dimensions.

**Figure 4:**
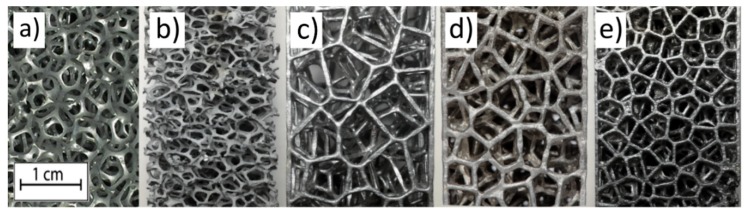
Metal foams object of this work: (**a**) m.pore®, (**b**) duocel®, (**c**) 5 pores-per-inch (PPI) designed, (**d**) 7 PPI designed, and (**e**) 10 PPI designed.

The accuracy of the designed process led to the achievement of samples with the same PPI numbers as the CAD model. The PPI number is a statistical classification, as it is strongly influenced by the manufacturing process. Mainly, it affects the distribution and the shape of the cavities within the foam. The two commercial foams exploited in this paper were both characterized by a PPI number equal to 10, but this is only a value to catalogue the porosity of commercial foams. However, they highlight a different morphology from the cast 10 PPI specimen. In the indirect additive manufacturing (I-AM) developed, the focus is on the modeling and not on the process parameters, as they provide limited control of the structure. The overall quality of the commercial products available in the market may be obtained only by means of a statistical approach. Figure 4 shows the metal foams object of this work. The comparison between the pictures c) and e) of Figure 4 shows how linear the ratio of 1:2 is, as expected from the observation of 5 and 10 PPI foams.

**Figure 5:**
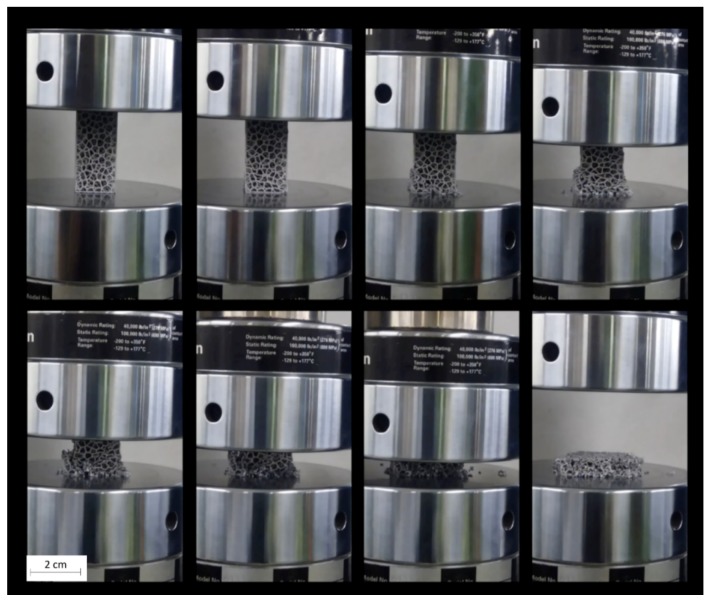
Frames from compression test of 10 PPI designed metal foam specimen.

The compression tests of the same foam type have the same mechanical behavior, highlighting the repeatability of the characteristics obtained. During a compression test, three areas can be individuated: an elastic, a plateau, and an ending zone where the specimen failure occurs. Figure 5 shows a designed specimen of 10 PPI during the compression test. In the second frame of the first row, there is the end of the elastic stretch, while in the second frame of the second row there is phenomenon of densification of the cells. Plastic deformation and fracture of cell wall progress simultaneously to a distinct peak followed by a small stress drop. This behavior is evident in the sequence frames. The densification phenomenon involves an extremity of the sample with a slow propagation on the rest of the specimen until reaching a deformation of 50%. After the collapse of the structure, densification involves the whole morphology, and the stress/strain curve starts again with a significant increase.

**Figure 6:**
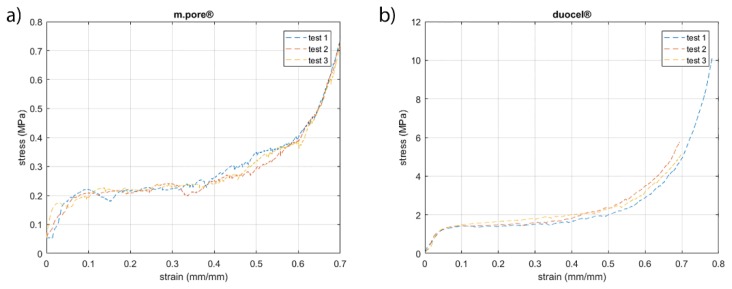
Stress-strain curves of commercial foams investigated. Respectively, (**a**) m.pore® and (**b**) duocel®.

**Figure 7:**
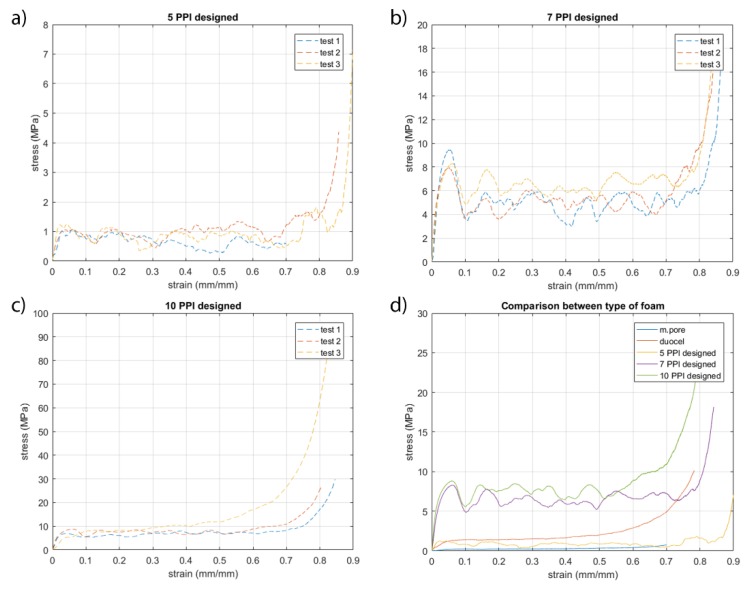
Stress-strain curves of designed foams. Respectively, (**a**) 5 PPI designed, (**b**) 7 PPI designed, (**c**) 10 PPI designed, and (**d**) comparison between type of foams investigated.

Figure 6 and Figure 7 show the stress-strain curves. The curves obtained are in accordance with typical stress-strain curve of solid foams. The plateau is rather smooth and corresponds to progressive cell collapse by elastic buckling, plastic yielding, or brittle crushing. Figure 6 shows the stress-strain curves obtained from compression tests of commercial foams. The curves show a very slight dispersion of data between them. This is due to the fact that ligaments tend to compact without breaking up during breakage. This behavior is in agreement with the fact that both foams have very similar production processes that statistically exhibit replicable behavior. Figure 7 shows the stress-strain curves of designed foams and the comparison between different types of foams. The curves of the same structure are similar, as they are on the same stress value. However, few oscillations are evident and cause the breaking of different ligaments in the same level of deformation between different samples. Designed 5 and 7 PPI foams have large cells and very long ligaments; in these tests, the oscillations observed on the plateau are the results of breaking individual ligaments. The designed foams have a section of ligament constant along its axis. In contrast, commercial foams have a variable section of ligament, less in the mean section between the two ends and greater at the node. An additional possible explanation for this behavior is due to the surface obtained in the samples made. Ligaments show the profile of the layers created by the additive manufacturing process and reproduced by the casting process. Ten PPI samples show different results; this behavior is the consequence of morphology specimens. In fact, Figure 4 shows how the morphology of 10 PPI specimens are very different (a, b, e). In particular, the mechanical performances increase because the duocel® foam presents a structure closer than the m.pore® foam. In addition, designed 10 PPI foam shows a more constant section than the duocel® foam. Morphological characteristics allow one to obtain performances of different orders of magnitude, as shown in Table 2.

It was noted that all the specimens show a trend regarding the energy of deformation and their relative density that is calculated as the ratio between foam density and metal density.

**Figure 8:**
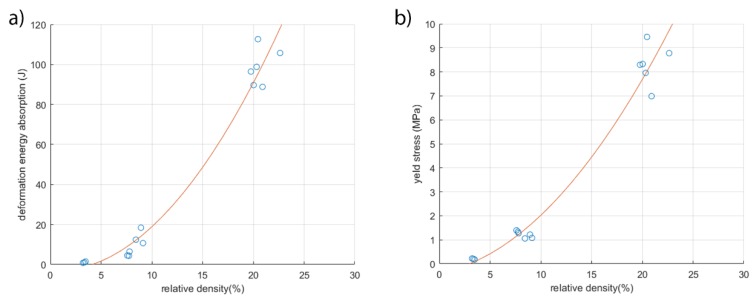
Respectively, (**a**) deformation energy absorbed and (**b**) yield strength as a function of the relative density of the specimens.

As highlighted in Figure 8, there is a second degree polynomial correlation between the deformation energy absorption and the relative density (R-square = 0.9674). This is an enhancement due to the improved resistance of the material. The same behavior occurs when investigating the yield stress (R-square = 0.9751). Improving the relative density led to increasing deformation energy and yield stress, as it involves an increased working area section.

The comparison between the duocel® foam and 5 PPI designed foam showed that, although the samples present a similar value of relative density, the deformation energy absorption of designed foam is superior to commercial foam. This consideration shows the influence of the morphology on the mechanic characteristics. In fact, the foams with similar values of relative density show different geometrical characteristics of ligaments. These specimens display a similar density value, but the designed foam exhibits twice the deformation energy absorption value of the commercial foam. These results highlight the importance of the shape control functionalized to the specific application.

**Figure 9:**
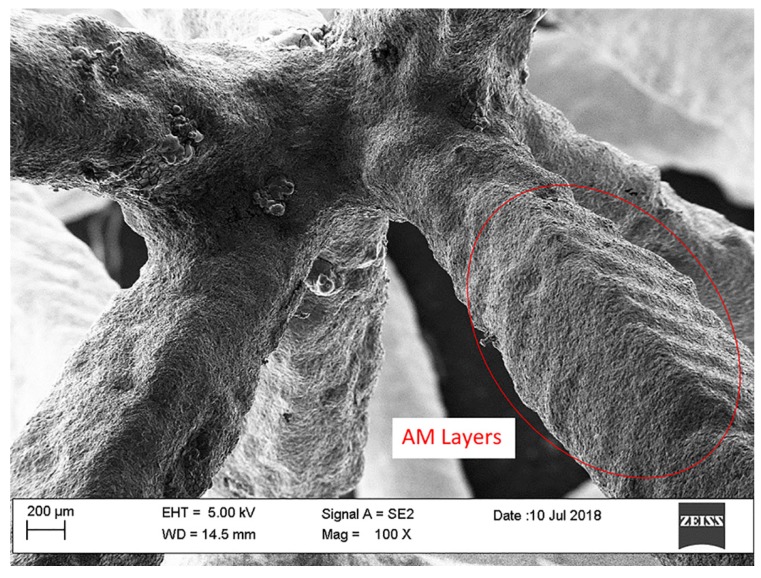
SEM image of 5 PPI-designed metal foam.

It has been noted that the specimens produced in this work during the compression test showed the separation of ligaments. A Scanning Electron Microscopy (SEM) analysis was performed; Figure 9 displays a SEM image of a node of ligaments of 5 PPI designed metal foam. As highlighted in Figure 9, printed layers were reproduced exactly by foundry process on ligament morphology. The morphology characteristics highlighted are associated with resin foam printing process.

## 4. Conclusions

This paper proposes the design and the production of metal foams with a completely customized structure. An algorithm for the Voronoi tessellation of a specific region was implemented and led to the realization of the cellular structure typical of metal foams.

The CAD files created were exploited for the production of the casting models by additive manufacturing.

Comparing the CAD models with 3D models and cast components highlights the high accuracy of this indirect additive manufacturing process in the production of metal foams. In contrast to the commercial foams, it is possible to design and therefore produce metal foam featuring PPI value and ligaments morphology in accordance with design needs. This allows for the design of components dedicated to specific applications. Quasi-static compression tests performed on the commercial foams and samples produced highlight the second-degree correlation between the density and deformation energy absorption of the specimens. However, it is possible to evaluate the structural improvement for the increase of the deformation energy. In particular, the 5 PPI designed structure, despite comparable value of relative density, exhibits twice the deformation energy absorption value compared to the 10 PPI duocel® commercial foam.

## Figures and Tables

**Table 1:** Number of pores of foams designed in this work.

PPI	Pores Per Centimeter	Specific Pores (pores/cm^3^)	Number of Pores
5	1.968	7.628	183
7	2.756	20.931	502
10	3.937	61.024	1,465

**Table 2:** Results of compression tests.

Typology Foam	Specimen	Relative Density (%)	Y Strength (MPa)	Y Strain	Densification Strength (MPa)	Densification Strain	Deformation Energy Absorption (J)
m.pore®	1	3.217	0.221	0.094	0.295	0.447	0.795
m.pore®	2	3.351	0.207	0.091	0.279	0.481	1.069
m.pore®	3	3.475	0.175	0.030	0.265	0.460	1.491
duocel®	1	7.804	1.276	0.059	1.993	0.499	6.494
duocel®	2	7.592	1.397	0.072	1.900	0.411	4.521
duocel®	3	7.745	1.337	0.058	2.087	0.454	4.394
5 PPI designed	1	9.133	1.080	0.064	0.589	0.698	10.682
5 PPI designed	2	8.430	1.057	0.040	1.260	0.705	12.419
5 PPI designed	3	8.932	1.220	0.022	1.232	0.846	18.403
7 PPI designed	1	20.462	9.457	0.052	9.532	0.835	112.677
7 PPI designed	2	20.326	7.955	0.051	7.907	0.764	98.823
7 PPI designed	3	19.769	8.295	0.060	7.730	0.780	96.485
10 PPI designed	1	20.913	6.985	0.040	11.937	0.507	88.822
10 PPI designed	2	22.643	8.780	0.057	9.777	0.636	105.762
10 PPI designed	3	20.036	8.327	0.125	7.720	0.666	89.701

## References

[1] Gibson I., Rosen D., Stucker B. (2015). Additive Manufacturing Technologies.

[2] Turner B.N., Strong R., Gold S.A. (2014). A review of melt extrusion additive manufacturing processes: I. Process design and modeling. Rapid Prototyp. J..

[3] Zein I., Hutmacher D.W., Tan K.C., Teoh S.H. (2002). Fused deposition modeling of novel scaffold architectures for tissue engineering applications. Biomaterials.

[4] Anitha R., Arunachalam S., Radhakrishnan P. (2001). Critical parameters influencing the quality of prototypes in fused deposition modelling. J. Mater. Process. Technol..

[5] Minetola P., Iuliano L., Marchiandi G. (2016). Benchmarking of FDM Machines through Part Quality Using IT Grades. Procedia CIRP.

[6] Ngo T.D., Kashani A., Imbalzano G., Nguyen K.T.Q., Hui D. (2018). Additive manufacturing (3D printing): A review of materials, methods, applications and challenges. Compos. Part B Eng..

[7] Herzog D., Seyda V., Wycisk E., Emmelmann C. (2016). Additive manufacturing of metals. Acta Mater..

[8] Antenucci A., Guarino S., Tagliaferri V., Ucciardello N. (2015). Electro-deposition of graphene on aluminium open cell metal foams. Mater. Des..

[9] Ju J., Summers J.D., Ziegert J., Fadel G. (2012). Design of Honeycombs for Modulus and Yield Strain in Shear. J. Eng. Mater. Technol..

[10] Gibson L.J., Ashby M.F. (1990). Cellular Solids: Structure and Properties.

[11] Simoncini A., Ucciardello N., Tagliaferri V. (2016). Thermal and mechanical improvement of aluminum open-cells foams through electrodeposition of copper and graphene. Manuf. Rev..

[12] Devivier C., Tagliaferri V., Trovalusci F., Ucciardello N. (2015). Mechanical characterization of open cell aluminium foams reinforced by nickel electro-deposition. Mater. Des..

[13] Antenucci A., Guarino S., Tagliaferri V., Ucciardello N. (2014). Improvement of the mechanical and thermal characteristics of open cell aluminum foams by the electrodeposition of Cu. Mater. Des..

[14] Ju J., Kim D.M., Kim K. (2012). Flexible cellular solid spokes of a non-pneumatic tire. Compos. Struct..

[15] Heo H., Ju J., Kim D.M. (2013). Compliant cellular structures: Application to a passive morphing airfoil. Compos. Struct..

[16] Kim K., Ju J., Kim D.-M. (2013). Porous materials with high negative Poisson’s ratios—A mechanism based material design. Smart Mater. Struct..

[17] Schultz J., Griese D., Ju J., Shankar P., Summers J.D., Thompson L. (2012). Design of Honeycomb Mesostructures for Crushing Energy Absorption. J. Mech. Des..

[18] Altenbach H., Öchsner A. (2010). Cism International Centre for Mechanical Sciences: Cellular and Porous Materials in Structures and Processes.

[19] Phani A.S., Woodhouse J., Fleck N.A. (2006). Wave propagation in two-dimensional periodic lattices. J. Acoust. Soc. Am..

[20] Guarino S., Di Ilio G., Venettacci S. (2017). Influence of thermal contact resistance of aluminum foams in forced convection: Experimental analysis. Materials.

[21] Barbieri M., Di Ilio G., Patanè F., Bella G. (2017). Experimental investigation on buoyancy-induced convection in aluminum metal foams [Étude expérimentale sur la convection provoquée par poussée hydrostatique dans les mousses métalliques en aluminium]. Int. J. Refrig..

[22] Guarino S., Rubino G., Tagliaferri V., Ucciardello N. (2015). Thermal behavior of open cell aluminum foams in forced air: Experimental analysis. Meas. J. Int. Meas. Confed..

[23] Almonti D., Ucciardello N. (2019). Design and Thermal Comparison of Random Structures Realized by Indirect Additive Manufacturing. Materials.

[24] Baiocco G., Tagliaferri V., Ucciardello N. (2017). Neural Networks Implementation for Analysis and Control of Heat Exchange Process in a Metal Foam Prototypal Device. Procedia CIRP.

[25] Banhart J. (2001). Manufacture, characterisation and application of cellular metals and metal foams. Prog. Mater. Sci..

[26] Ashby M. (2000). Metal Foams: A Design Guide.

[27] Hutter C., Büchi D., Zuber V., Rudolf von Rohr P. (2011). Heat transfer in metal foams and designed porous media. Chem. Eng. Sci..

[28] Nowacki J., Moraniec K. (2015). Welding of metallic AlSi foams and AlSi-SiC composite foams. Arch. Civ. Mech. Eng..

[29] Kuruneru S.T.W., Sauret E., Saha S.C., Gu Y.T. (2016). Numerical investigation of the temporal evolution of particulate fouling in metal foams for air-cooled heat exchangers. Appl. Energy.

[30] Bagcl O., Dukhan N., Ozdemir M., Kavurmacloglu L.A. (2016). Experimental heat transfer due to oscillating water flow in open-cell metal foam. Int. J. Therm. Sci..

[31] Bağcı Ö., Dukhan N. (2016). Experimental hydrodynamics of high-porosity metal foam: Effect of pore density. Int. J. Heat Mass Transf..

[32] Almonti D., Simoncini M., Tagliaferri V., Ucciardello N. (2018). Electro-deposition of graphene nanoplatelets on CPU cooler—Experimental and numerical investigation. Mater. Manuf. Process..

[33] Almonti D., Ucciardello N. (2019). Improvement of thermal properties of micro head engine electroplated by graphene: Experimental and thermal simulation. Mater. Manuf. Process..

[34] Shen B., Yan H., Sunden B., Xue H., Xie G. (2017). Forced convection and heat transfer of water-cooled microchannel heat sinks with various structured metal foams. Int. J. Heat Mass Transf..

[35] Wang Y., Tian H., Shu G., Yu G., Ma X., Li X. (2017). Simulation and Optimization of Metal-foam Tube Banks for Heat Transfer Enhancement of Exhaust Heat Exchangers. Energy Procedia.

